# Supplementary material for: Vaccination and Factors Related to the Clinical Outcome of COVID-19 in Healthcare Workers—A Romanian Front-Line Hospital’s Experience
Source: Vaccines (Basel). 2023 Apr 25;11(5):899. doi: 10.3390/vaccines11050899 (PMC10222041; doi:10.3390/vaccines11050899)
Supplement: Supplementary file 1 [file vaccines-11-00899-s001.zip › vaccines-2339159-supplementary.pdf]

Supplementary Material

Table S1. COVID-19 cases in HCWs and general population, April 2020 – Dec 2021, Romania (the extracted data from National Center for Surveillance and Disease Control weekly reports) .

Year	Month	Week	Cumulative number of cases in HCWs	Weekly cases in HCWs	Cumulative number of cases in Roumanian population	Weekly cases in Roumanian population
2020	APR	30.03-05.04.2020	662		4068	
2020	APR	06.04-12-04.2020	903	241	6633	2565
2020	APR	13.04-19.04.2020	1213	310	8936	2303
2020	APR	20.04-26.04.2020	1286	73	11313	2377
2020	MAY	27.04-03.05.2020	1771	485	13552	2239
2020	MAY	04.05-10.05.2020	2264	493	15501	1949
2020	MAY	11.05-17.05.2020	2601	337	16895	1394
2020	MAY	18.05-24.05.2020	2763	162	18283	1388
2020	MAY	25.05-31.05.2020	2865	102	19400	1117
2020	JUN	01.06-07.06.2020	2909	44	20605	1205
2020	JUN	08.06-14.06.2020	3000	91	22160	1555
2020	JUN	15.06-21.06.2020	3149	149	24284	2124
2020	JUN	22.06-28.06.2020	3267	118	26582	2298
2020	JUL	29.06-05.07.2020	3335	68	29224	2642
2020	JUL	06.07-12.07.2020	3422	87	32946	3722
2020	JUL	13.07-19.07.2020	3567	145	38139	5193
2020	JUL	20.07-26.07.2020	3714	147	45902	7763
2020	AUG	27.07-02.08.2020	3849	135	54009	8107
2020	AUG	03.08-09.08.2020	3992	143	62547	8538
2020	AUG	10.08-16.08.2020	4134	142	71194	8647
2020	AUG	17.08-23.08.2020	4266	132	79330	8136
2020	AUG	24.08-30.08.2020	4378	112	87549	8219
2020	SEPT	31.08-06.09.2020	4673	295	95897	8348
2020	SEPT	07.09-13.09.2020	4683	10	104187	8290
2020	SEPT	14.09-20.09.2020	4776	93	113589	9402
2020	SEPT	21.09-27.09.2020	4922	146	123944	10355
2020	OCT	28.09-04.10.2020	4997	75	137491	13547
2020	OCT	05.10-11.10.2020	5346	349	157352	19861
2020	OCT	12.10-18.10.2020	5623	277	182854	25502
2020	OCT	19.10-25.10.2020	6327	704	250704	67850
2020	OCT	26.10-01.11.2020	6636	309	306991	56287
2020	NOV	02.11-08.11.2020	6955	319	365212	58221
2020	NOV	09.11-15.11.2020	7370	415	422852	57640
2020	NOV	16.11-22.11.2020	7842	472	475362	52510
2020	NOV	23.11-29.11.2020	8154	312	517236	41874
2020	DEC	30.11-06.12.2020	8942	788	559587	42351
2020	DEC	07.12-13.12.2020	8951	9	593783	34196
2020	DEC	14.12-20.12.2020	9312	361	618429	24646
2020	DEC	21.12-27.12.2020	9803	491	643559	25130
2021	JAN	04.01-10.01.2021	10338	535	673271	29712
2021	JAN	11.01-17.01.2021	10646	308	695153	21882

2021	JAN	18.01-24.01.2021	10812	166	712561	17408
2021	JAN	25.01-31.01.2021	11021	209	730056	17495
2021	FEBR	01.02-07.02.2021	11208	187	746637	16581
2021	FEBR	08.02-14.02.2021	12368	1160	763294	16657
2021	FEBR	15.02-21.02.2021	12523	155	781329	18035
2021	FEBR	22.02-28.02.2021	12607	84	804090	22761
2021	MAR	01.03-07.03.2021	12725	118	830563	26473
2021	MAR	08.03-14.02.2021	13087	362	862681	32118
2021	MAR	15.03-21.03.2021	13093	6	900858	38177
2021	MAR	22.03-28.03.2021	13264	171	904443	3585
2021	APR	29.03-04.04.2021	13492	228	977986	73543
2021	APR	05.04-11.04.2021	13646	154	1008490	30504
2021	APR	12.04-18.04.2021	13729	83	1031072	22582
2021	APR	19.04-25.04.2021	13799	70	1047520	16448
2021	APR	26.04-02.05.2021	13861	62	1050337	2817
2021	MAY	03.05-09.05.2021	13925	64	1066731	16394
2021	MAY	10.05-16.05.2021	13951	26	1072291	5560
2021	MAY	17.05-23.05.2021	13969	18	1075773	3482
2021	MAY	24.05-30.05.2021	13995	26	1077735	1962
2021	JUN	31.05-06.06.2021	14027	32	1078952	1217
2021	JUN	07.06-13.06.2021	14122	95	1079776	824
2021	JUN	14.06-20.06.2021	14142	20	1080282	506
2021	JUN	21.06-27.06.2021	14221	79	1080667	385
2021	JUL	28.06-04.07.2021	14265	44	1080979	312
2021	JUL	05.07-11.07.2021	14314	49	1081275	296
2021	JUL	12.07-18.07.2021	14379	65	1081678	403
2021	JUL	19.07-25.07.2021	14405	26	1082376	698
2021	AUG	26.07-01.08.2021	14432	27	1083478	1102
2021	AUG	02.08-08.08.2021	14476	44	1085100	1622
2021	AUG	09.08-15.08.2021	14495	19	1087509	2409
2021	AUG	16.08-22.08.2021	14523	28	1091340	3831
2021	AUG	23.08-29.08.2021	14572	49	1097452	6112
2021	SEPT	30.08-05.09.2021	14653	81	1107043	9591
2021	SEPT	06.09-12.09.2021	14757	104	1122654	15611
2021	SEPT	13.09-19.09.2021	15006	249	1152052	29398
2021	SEPT	20.09-26.09.2021	15338	332	1199761	47709
2021	OCT	27.09-03.10.2021	15527	189	1274119	74358
2021	OCT	04.10-10.10.2021	15765	238	1365788	91669
2021	OCT	11.10-17.10.2021	16418	653	1467401	101613
2021	OCT	18.10-24.10.2021	16754	336	1571115	103714
2021	OCT	25.10-31.10.2021	16988	234	1655024	83909
2021	NOV	01.11-07.11.2021	17182	194	1711137	56113
2021	NOV	08.11-14.11.2021	17321	139	1744440	33303
2021	NOV	15.11-21.11.2021	17392	71	1764251	19811
2021	NOV	22.11-28.11.2021	17441	49	1778045	13794
2021	DEC	29.11-05.12.2021	17479	38	1786839	8794
2021	DEC	06.12-12.12.2021	17508	29	1793643	6804
2021	DEC	13.12-19.12.2021	17538	30	1798629	4986
2021	DEC	20.12-16.12.2021	17556	18	1803311	4682
2021	DEC	27.12-02.01.2022	17584	28	1813056	9745

Source: National Center for Surveillance and Control of Communicable Disease. COVID-19 Confirmed Cases Analysis. Available online: https://www.cnscbt.ro/index.php/analiza-cazuri-confirmate-covid19 (accessed on 15 March 2023).
